# Ginsenoside Rg3 Inhibits Migration and Invasion of Nasopharyngeal Carcinoma Cells and Suppresses Epithelial Mesenchymal Transition

**DOI:** 10.1155/2019/8407683

**Published:** 2019-02-24

**Authors:** Dingkun Wang, Cheng Wu, Dongbo Liu, Linli Zhang, Guoxian Long, Guangyuan Hu, Wei Sun

**Affiliations:** ^1^Department of Integrated Traditional Chinese and Western Medicine, Tongji Hospital, Tongji Medical College, Huazhong University of Science and Technology, Wuhan 430030, China; ^2^Department of Oncology, Tongji Hospital, Tongji Medical College, Huazhong University of Science and Technology, Wuhan 430030, China

## Abstract

Nasopharyngeal carcinoma (NPC) is a highly invasive and metastatic head and neck cancer. Distant metastasis becomes the predominant mode of treatment failure in NPC patients. Ginsenoside Rg3 (Rg3), an active pharmaceutical component extracted from traditional Chinese medicine ginseng, shows antitumor effects in various cancers. In this study, we aimed to determine whether Rg3 inhibits the migration and invasion activity of NPC cells and to explore the possible mechanisms. Our results revealed that Rg3 hampers cell migration and invasion in both HNE1 and CNE2 cell lines. A reduced level of matrix metalloproteinase-2 (MMP-2) and MMP-9 was induced by Rg3 treatment. In addition, Rg3 significantly altered the expression of epithelial mesenchymal transition (EMT) markers with increased E-cadherin but decreased Vimentin and N-cadherin expression. Transforming growth factor *β*- (TGF-*β*-) induced morphological transition and marker proteins change of EMT were reversed by Rg3. What is more, Rg3 suppressed the expression of EMT-related transcription factors, especially the Zinc Finger E-Box Binding Homeobox 1 (ZEB1). In summary, our data suggested that Rg3 could inhibit migration and invasion of NPC cells. This effect of Rg3 might be mediated through regulating MMP-2 and MMP-9 expressions and suppressing EMT. Thus, Rg3 may be a potentially effective agent for the treatment of NPC.

## 1. Introduction

Nasopharyngeal carcinoma (NPC) is the most common head and neck cancer in Southern China [1]. It shows highly invasive and metastatic feature with approximately 75% of patients present with regional lymph node metastasis and 10% present with distant metastasis at the time of diagnosis [2, 3]. Due to its anatomical location and radiosensitivity, radiotherapy remains the primary therapeutic approach for this disease [4]. Intensity-modulated radiotherapy (IMRT), an advanced form of radiation technique, is able to deliver higher dose to the tumor and lower dose to the surrounding normal tissues. In recent years, IMRT had been widely adopted in cancer treatment and its technical advantages achieved excellent local and regional controls in NPC patients [5–7]. The failure pattern of NPC tends to change from locoregional recurrence and distant metastasis to predominantly distant metastasis. Therefore, a better understanding of the molecular mechanisms of NPC invasion and metastasis is urgently needed and helpful for improving the survival of NPC patients.

Although comprehensive treatments are employed for cancer, the final effect is still not satisfactory. Therefore, the development of novel agents to treat tumor is important. It is now generally accepted that natural products have unique therapeutic effect on a variety of cancers. Ginsenosides, active pharmaceutical components extracted from the traditional Chinese medicine ginseng, play important roles in the prevention and treatment of cancers [8–12]. Among them, ginsenoside Rg3 (Rg3) is attracting increasing attention as it has multiple anticancer effects, including the induction of apoptosis, the inhibition of proliferation, the restrain of metastasis and angiogenesis, and the promotion of immunity [8]. It is reported that Rg3 exhibits antitumor effects in many kinds of cancers [13–15]. In addition, Rg3 can be a beneficial supplement for conventional cancer therapies. The combined administration of chemotherapeutic agents and Rg3 is more effective than either one being adopted alone [16–18]. In the year of 2002, Rg3 was approved by the Chinese Food and Drug Administration (CFDA) for treating nonsmall cell lung cancer [17]. These studies indicated that Rg3 may play a role in the prevention and treatment of cancers.

Tumor invasion and metastasis is a complex process through which cancer cells spread from a primary site to other distant parts of the body. It results from multiple steps which include the degradation of surrounding extracellular matrix (ECM). Matrix metalloproteinases (MMPs) are a family of proteinases whose enzymatic activity is directed against ECM [19]. Several studies reported that Rg3-induced suppression of MMP-9 and MMP-13 is associated with decreased migration and invasive capacity of cancer cells [20–22]. Epithelial mesenchymal transition (EMT), a process by which epithelial cells differentiate into motile mesenchymal cells, is also an important mechanism involved in cancer metastasis [23, 24]. Recently, mounting evidence indicated that Rg3 could inhibit EMT in various cancers. Transforming growth factor *β*- (TGF-*β*-) induced EMT and cell invasion in lung cancer can be suppressed by Rg3 [25]. In ovarian cancer, Rg3 inhibits EMT by reducing hypoxia-inducible factor 1 (HIF-1) [26]. These studies indicated that Rg3 is a potential therapeutic agent to control tumor invasion and metastasis.

However, it is still not clear whether Rg3 exerts antitumor effect in NPC. The exact mechanism of Rg3 inhibiting invasion and metastasis is also unknown. Thus, we performed this study to evaluate the role of Rg3 in inhibiting migration and invasion of NPC cells and to explore the possible mechanism for the first time.

## 2. Materials and Methods

### 2.1. Reagents

The 20(S)-Rg3 was provided by the Yiyan Biological Technology Company (Shanghai, China). Rg3 was dissolved in dimethyl sulfoxide (DMSO) in a 200mg/ml stock solution and stored at -20°C and diluted with fresh culture medium before use. An equal volume of DMSO (final concentration <0.1%) was added to the controls. The recombinant human TGF-*β* was purchased from PeproTech (Rocky Hill, NJ, USA). Primary monoclonal antibodies including rabbit anti-human MMP-2, MMP-9, E-cadherin, N-cadherin, Vimentin, Snail, Slug, Twist, Zinc Finger E-Box Binding Homeobox 1 (ZEB1), and glyceraldehyde-3-phosphate dehydrogenase (GAPDH) antibodies were purchased from Cell Signaling Technology (Danvers, MA, USA). The horseradish peroxidase-conjugated secondary antibodies were obtained from Santa Cruz Biotechnology (Santa Cruz, CA, USA). The ECM gel (E1270) was obtained from Sigma (St. Louis, MO, USA).

### 2.2. Cell Lines Culture

The human NPC cell lines, including HNE1 and CNE2, were obtained from the Cancer Research Institute of Central South University (Changsha, China) [27, 28]. They were cultured in RPMI-1640 medium containing 10% fetal bovine serum and incubated at 37°C in a humidified atmosphere of 5% CO2.

### 2.3. Reverse Transcription-Polymerase Chain Reaction (RT-PCR)

Cells were harvested and washed with phosphate buffered saline (PBS). RNA was extracted from cells using the RNAiso Plus kit (Takara Biotechnology, Dalian, China) according to the manufacturer's instructions. Then the first strand of cDNA was reversed using the PrimeScriptTM 1st Strand cDNA Synthesis Kit (Takara Biotechnology, Dalian, China). The RT-PCR was run in the 7900HT Fast Real-Time PCR System (Applied Biosystem, California, USA) and detected by using SYBR Select Master (Life Technologies, California, USA). The Primer Premier 5.0 software was used to design the primers for PCR.

### 2.4. Wound-Healing Migration Assay

Cells were seeded in 24-well plates and grown to confluent monolayer overnight for the wound-healing migration assay. The monolayer was scratched straightly with a 10 *μ*L pipette tip, and cellular debris was removed with PBS. Then the cells were incubated in serum-free medium containing different concentrations of Rg3 (0, 25, 50, and 100 *μ*g/ml). Migration was observed at the indicated times (0 and 24h) under a microscope. ImageJ analysis software was used to measure the migration distances.

### 2.5. Transwell Migration and Invasion Assays

Transwell migration and invasion assays were performed using transwell 24-well plates with 8*μ*m diameter filters (Corning, NY, USA). For migration assays, cells were placed into the upper chamber with the noncoated membrane. For invasion assays, filters were precoated with 40*μ*l of diluted ECM gel for 4h. Cells (1×10^5^) in 200*μ*l of serum-free medium containing different concentrations of Rg3 (0, 25, 50, and 100 *μ*g/ml) were placed in the upper chamber and 500*μ*l 10% newborn calf serum was placed in the lower chamber. After incubated for 20h, cells were fixed in methanol for 15 min and stained with 0.1% crystal violet for 15 min. Then cells on the upside of the filters were removed with a cotton swab, and cells on the underside were examined and counted under a microscope at ×200 magnification.

### 2.6. Western Blot Analysis

Following treatment, NPC cells were harvested with ice-cold PBS. Then proteins were extracted using a Total Protein Extraction Kit (Beyotime, Shanghai, China) at the indicated times. Protein concentrations were measured using bicinchoninic acid (BCA) protein assay kit according to the manufacturer's instructions (Beyotime, Shanghai, China). Then proteins were separated on SDS-PAGE and transferred to a polyvinylidene difluoride membrane (PVDF; Millipore, MA, USA). Membranes were blocked with 5% nonfat milk in TBST (Tris-buffered saline and 2.5% Tween-20) for 1 h at room temperature. Then each membrane was incubated with primary antibodies at 4°C for 20 h. After repeated washing with TBST, the membrane was incubated with secondary antibodies. Immunoblots were developed using enhanced chemiluminescence (ECL) detecting substrate (Pierce, Rockford, IL, USA). Images were captured with Micro-Chemi (DNR Bio-Imaging Systems, Israel), and the optical density of the bands was determined using ImageJ software. In this study, GAPDH was used as a cytoplasmic internal control.

### 2.7. Statistical Analysis

SPSS software (version 19.0; SPSS, Inc., Chicago, IL) was used to perform the statistical analyses. All experiments were repeated independently at least 3 times and all data were presented as mean ± standard deviations (SD). Statistical comparison was performed using Student's t test and* P *value less than 0.05 was considered statistically significant.

## 3. Results

### 3.1. Rg3 Inhibits the Migration Activity of NPC Cells

**Figure 1:**
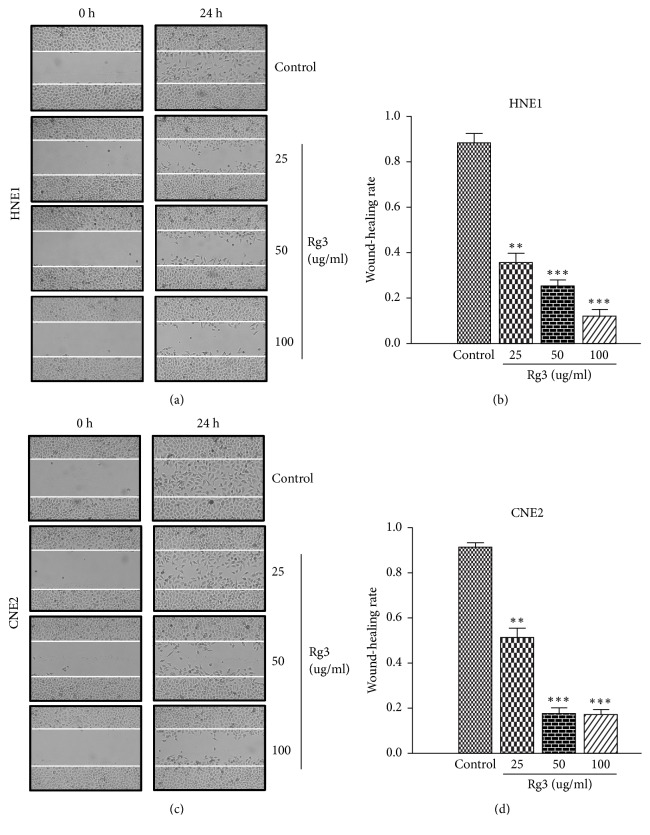
Effect of Rg3 on the wound-healing migration ability of HNE1 and CNE2 cells. (a, c) Monolayers of HNE1 and CNE2 cells were scraped and photographed in indicated times (×100; 0, 24 h). (b, d) Quantitative assessment of wound-healing rate in indicated times (24 h). Results are expressed as mean ± SD (n=3).  ^*∗∗*^P<0.01* vs* Control and  ^*∗∗∗*^P<0.001* vs* Control.

**Figure 2:**
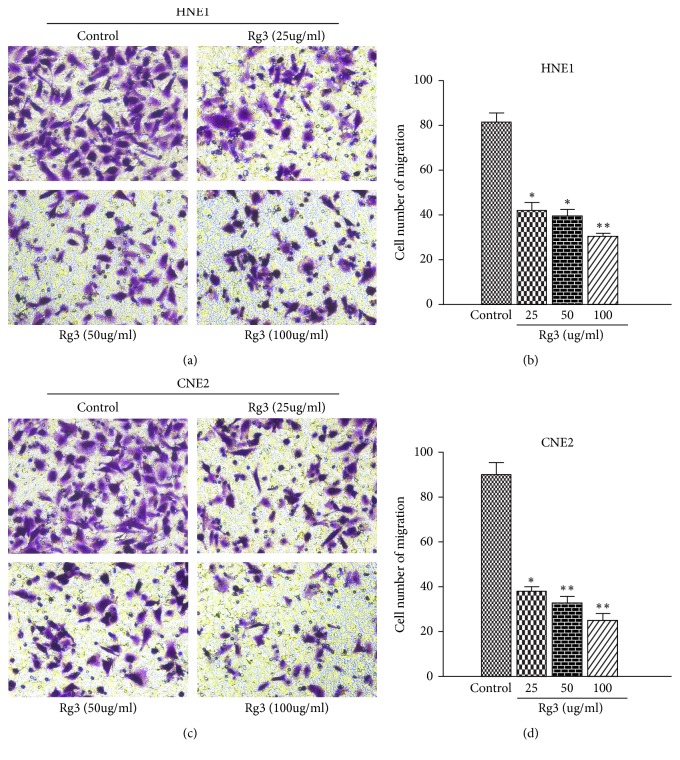
Effect of Rg3 on the transwell migration ability of HNE1 and CNE2 cells. (a, c) HNE1 and CNE2 cells were incubated with different doses of Rg3 for 24 h, and then cell migration was measured with the transwell assay (×200). (b, d) Quantitative assessments of the number of cells migrated to the underside chamber. Results are expressed as mean ± SD (n=3).  ^*∗*^P<0.05* vs* Control and  ^*∗∗*^P<0.01* vs* Control.

The inhibitory effect of Rg3 on NPC migration was examined by wound-healing migration assay and transwell migration assay. As showed in Figure 1, Rg3 significantly hampered wound-healing migration activity of NPC cells. When HNE1 and CNE2 cells were treated with Rg3 (100 *μ*g/ml) for 24h, the migration rate decreased to 12% and 15%, respectively. We also found that the transwell migration activity of NPC cells could be blocked by Rg3. When Rg3 was added to the upper compartment of the transwell plates, the number of cells that migrated to the underfilters decreased markedly (Figure 2). These data implied that Rg3 can decrease the migration ability of NPC cells obviously.

### 3.2. Rg3 Inhibits the Invasion Activity of NPC Cells

**Figure 3:**
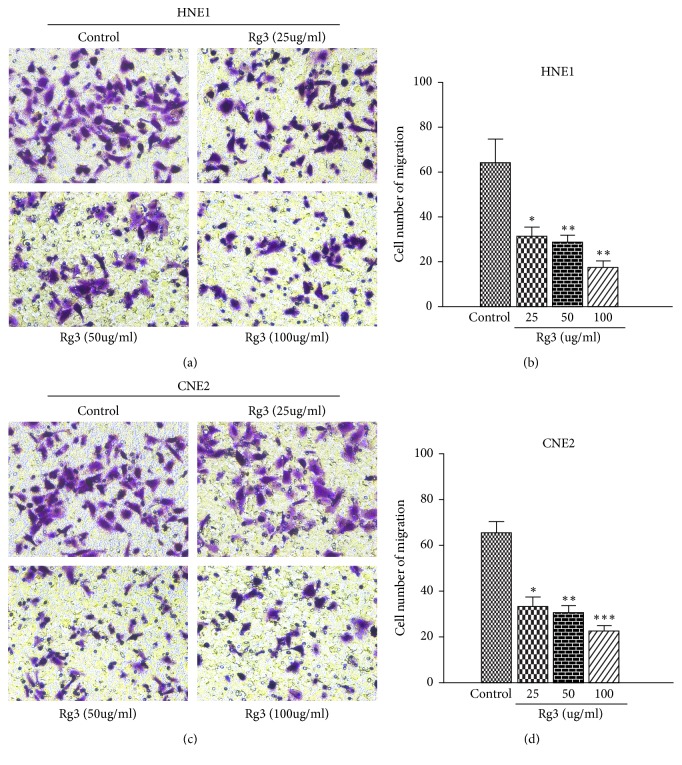
Effect of Rg3 on the invasion ability of HNE1 and CNE2 cells. (a, c) HNE1 and CNE2 cells were incubated with different doses of Rg3 for 24 h, and then cell migration was measured with the transwell assay (×200). (b, d) Quantitative assessments of the number of cells invaded to the underside chamber. Results are expressed as mean ± SD (n=3).  ^*∗*^P<0.05* vs* Control and  ^*∗∗*^P<0.01* vs* Control and  ^*∗∗∗*^P<0.001* vs* Control.

To examine the effect of Rg3 on NPC invasion, ECM gel was precoated to the upside of all filters. The invasion ability of HNE1 and CNE2 cells decreased when they were incubated with Rg3. As exhibited in Figure 3, the number of cells that penetrated into the lower chamber decreased significantly upon Rg3 treatment. These results demonstrated that Rg3 can attenuate the invasiveness of NPC cells.

### 3.3. Rg3 Reduces MMP-2 and MMP-9 Expressions in NPC Cells

**Figure 4:**
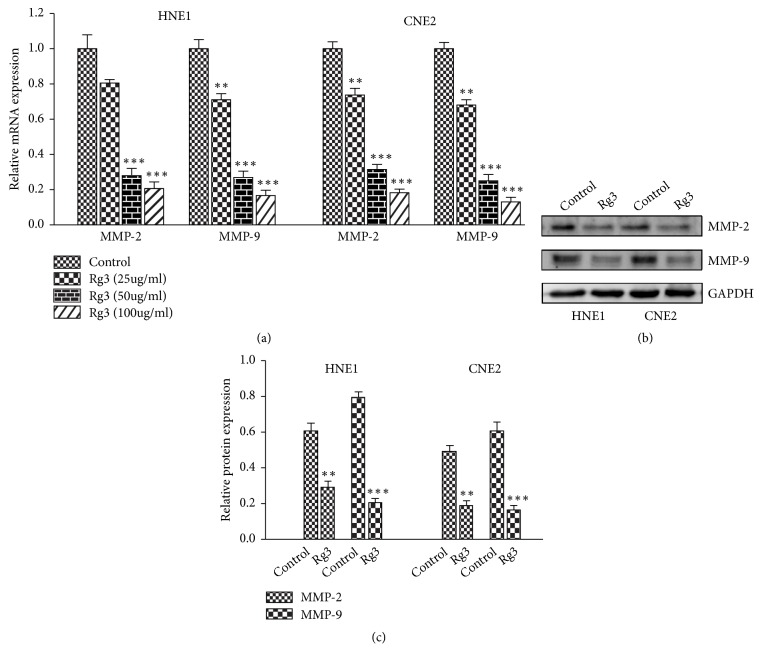
Effect of Rg3 on MMP-2 and MMP-9 expression in HNE1 and CNE2 cells. (a) mRNA levels of MMP-2 and MMP-9 were analysed by RT-PCR. (b) Protein levels of MMP-2 and MMP-9 were analysed by Western blot. (c) Relative protein expression of MMP-2 and MMP-9 in HNE1 and CNE2 cells. Results are expressed as mean ± SD (n=3).  ^*∗∗*^P<0.01* vs* Control and  ^*∗∗∗*^P<0.001* vs* Control.

MMP-2 and MMP-9 which selectively degrade the major component of ECM play a key role in the metastatic process [19]. Then we evaluated the influence of Rg3 on the expression of invasion-linked MMP-2 and MMP-9. The results of RT-PCR test showed that MMP-2 and MMP-9 decreased in dose-dependent manner upon Rg3 stimulation (Figure 4(a)). This inhibitory effect of Rg3 was further confirmed by Western blot when NPC cells were treated with Rg3 (100 *μ*g/ml) for 24h (Figures 4(b) and 4(c)). These results indicated that Rg3 may inhibit invasion of NPC cells through downregulating MMP-2 and MMP-9 expressions.

### 3.4. Rg3 Regulates EMT Markers in NPC Cells

**Figure 5:**
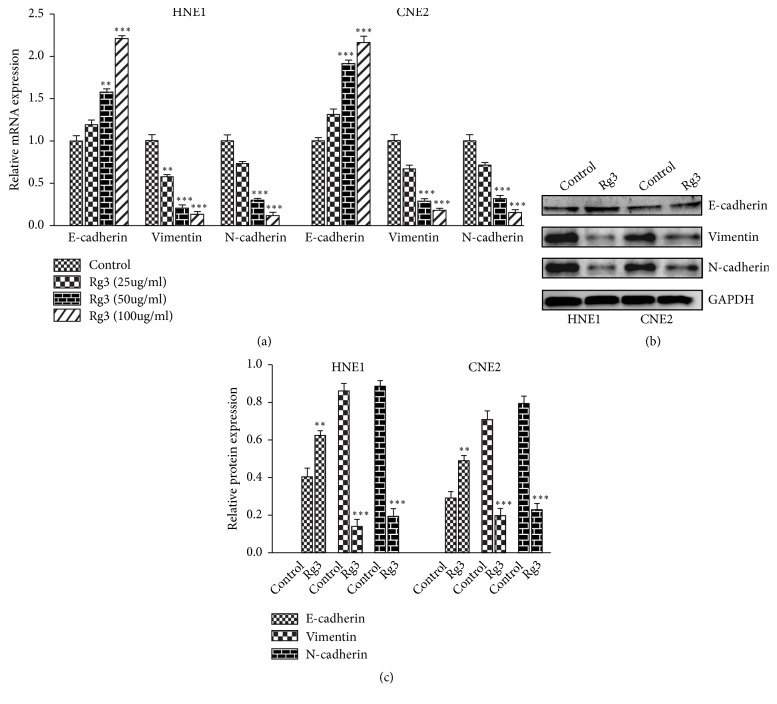
Effect of Rg3 on EMT markers expression in HNE1 and CNE2 cells. (a) mRNA levels of E-cadherin, N-cadherin, and Vimentin were analysed by RT-PCR. (b) Protein levels of E-cadherin, N-cadherin, and Vimentin were analysed by Western blot. (c) Relative protein expression of E-cadherin, N-cadherin, and Vimentin in HNE1 and CNE2 cells. Results are expressed as mean ± SD (n=3).  ^*∗∗*^P<0.01* vs* Control and  ^*∗∗∗*^P<0.001* vs* Control.

EMT is another important process involved in cancer metastasis. We next examined the influence of Rg3 on EMT markers. When treated with different concentrations of Rg3 (0, 25, 50, and 100 *μ*g/ml) for 24h, significant upregulation of E-cadherin and downregulation of Vimentin and N-cadherin were indicated by RT-PCR in HNE1 and CNE2 cells (Figure 5(a)). Then the impact of Rg3 on EMT markers was further verified by Western blot (Figures 5(b) and 5(c)). These data suggested that Rg3 is able to suppress EMT in a dose-dependent manner.

### 3.5. Rg3 Reverses TGF-*β*-Induced EMT

**Figure 6:**
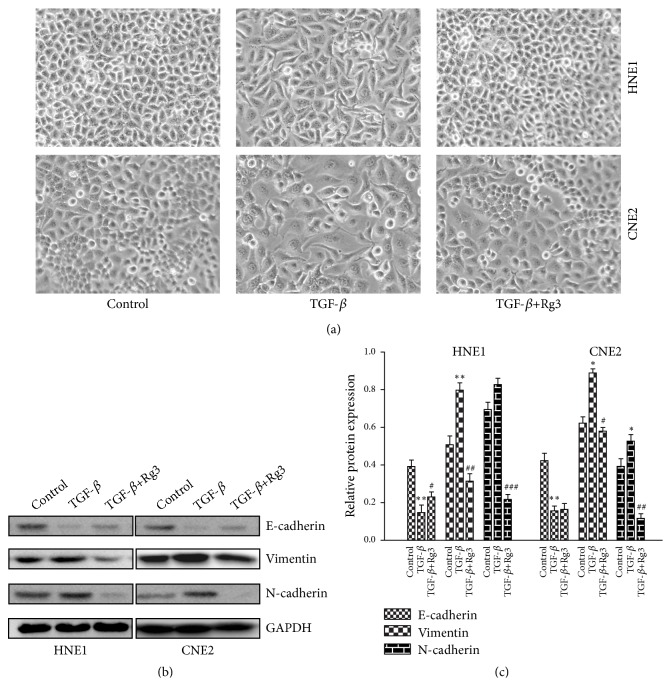
Effect of Rg3 on TGF-*β*-induced EMT in HNE1 and CNE2 cells. (a) Morphological changes of EMT in HNE1 and CNE2 cells were detected by invert microscope. (b) Protein levels of E-cadherin, N-cadherin, and Vimentin were analysed by Western blot. (c) Relative protein expression of E-cadherin, N-cadherin, and Vimentin in HNE1 and CNE2 cells. Results are expressed as mean ± SD (n=3).  ^*∗*^P<0.05* vs* Control, ^*∗∗*^P<0.01* vs* Control, ^#^P<0.05* vs* TGF-*β*, ^##^P<0.01* vs* TGF-*β*, and ^###^P<0.001* vs* TGF-*β*.

The front half of our study demonstrated that Rg3 can regulate the expression of EMT markers; thus we further tested whether Rg3 is able to reverse external stimulus-induced EMT in NPC cells. As shown in Figure 6(a), NPC cell lines presented with typical morphological transition of EMT after TGF-*β* (5ng/ml) stimulation. On the contrary, Rg3 hampered TGF-*β*-induced morphological change in NPC cells. In addition, TGF-*β* significantly altered EMT marker proteins with decreased E-cadherin but increased Vimentin and N-cadherin expression. This effect was also inhibited by Rg3 treatment (Figures 6(b) and 6(c)). These results indicated that Rg3 can reverse the process of EMT in NPC cells.

### 3.6. Rg3 Targets EMT-Related Transcription Factors in NPC Cells

**Figure 7:**
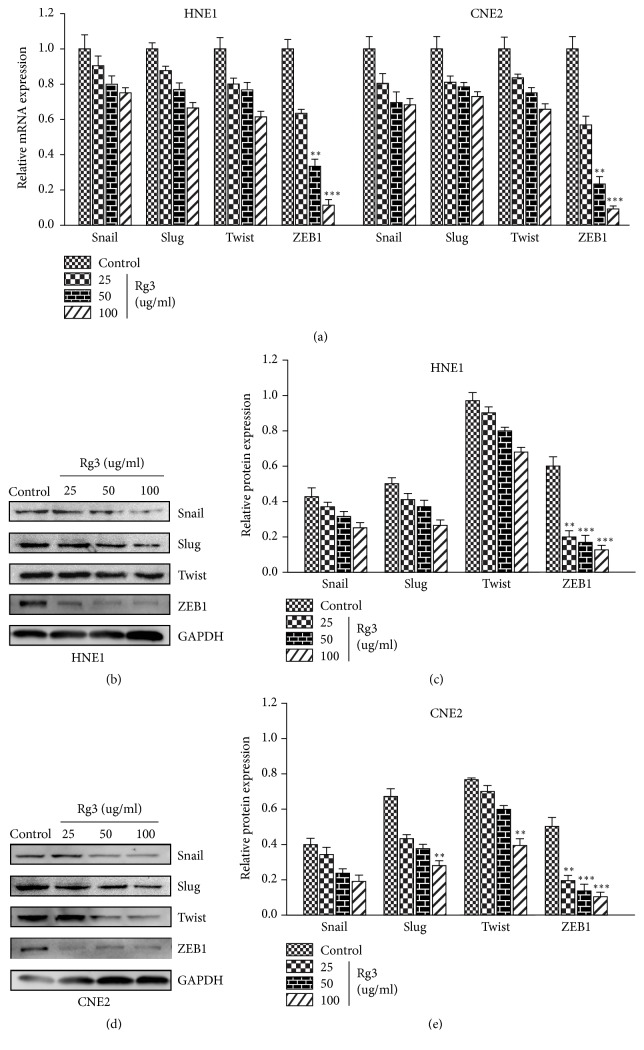
Effect of Rg3 on EMT-related transcription factors expression in HNE1 and CNE2 cells. (a) mRNA levels of Snail, Slug, Twist, and ZEB1 were analysed by RT-PCR. (b, d) Protein levels of Snail, Slug, Twist, and ZEB1 were analysed by Western blot. (c, e) Relative protein expression of Snail, Slug, Twist, and ZEB1 in HNE1 and CNE2 cells. Results are expressed as mean ± SD (n=3).  ^*∗∗*^P<0.01* vs* Control and  ^*∗∗∗*^P<0.001* vs* Control.

As EMT is driven by specific EMT-related transcription factors (EMT-TFs) [29], we hypothesized that Rg3 may suppress EMT in NPC cells by targeting EMT-TFs. After treatment with different concentrations of Rg3 (0, 25, 50, and 100 *μ*g/ml) for 24h, the mRNA level of several EMT-TFs was quantified. As shown, Snail, Slug, and Twist decreased slightly while ZEB1 declined markedly upon Rg3 stimulation (Figure 7(a)). The suppressive effect of Rg3 on Snail, Slug, and Twist proteins was also weak when they were tested by Western blot. But for ZEB1, Rg3 exerts a pronounced inhibitory effect on it (Figures 7(b), 7(c), 7(d), and 7(e)). Our data supported the idea that Rg3 may target EMT-TFs and cause the suppression of EMT in NPC cells.

## 4. Discussion

Currently, radiotherapy is the primary treatment for NPC. With the improvements in radiotherapy techniques, local recurrence substantially decreases and distant metastasis becomes the main cause of treatment failure. Thus, identifying drug agents to prevent and inhibit NPC metastasis is helpful for improving the survival of NPC patients. In this study, we provided the evidence that Rg3 can inhibit the migration and invasion ability of NPC cells as well as the process of EMT.

Cancer management always involves several conventional modalities which include surgery, radiotherapy, chemotherapy, and targeted therapy. Although surgery and radiotherapy are effective in the treatment of local tumors, they are often limited by tumor metastasis. Chemotherapy is the most commonly used treatment option for cancer, but it causes multiple side effects. Targeted therapy became a research focus and provided a new method to treat tumors in recent years. However, targeted drugs are expensive and always have the issue of acquired drug resistance. Therefore, developing novel agents which can effectively inhibit cancer without serious side effects is important. Traditional Chinese medicines have been shown to provide a rich resource for the identification of anticancer drugs. Rg3, an active component derived from ginseng, has been reported to possess antitumor effects in various cancers [8]. However, whether Rg3 exerts antitumor effect in NPC had not been evaluated up to now. In addition, the effect of Rg3 on tumor behaviors may depend on tumor cell types. Thus, we tested the inhibitory effect of Rg3 in NPC cells at the start of this study. We observed that Rg3 hampered migration activity of NPC cells. In the subsequent experiments, we found that cell number of invasion decreased obviously upon Rg3 stimulation. Based on these findings, we considered that Rg3 is able to inhibit migration and invasion of NPC cells and Rg3 may be a potential antimetastasis agent. Shen-Yi capsule, a finished pharmaceutical product adapted for oral delivery of Rg3, has been approved by the CFDA for cancer therapy [17]. Future clinical researches using Shen-Yi capsule may help us to determine the value of clinical application of Rg3 in NPC patients.

MMPs, especially MMP-2 and MMP-9, play crucial roles in tumor invasion and metastasis. Some previous studies have indicated that Rg3 is able to suppress the expression of MMPs. The Rg3-induced downregulation of MMP-9 is associated with the decreased invasive capacity of ovarian cancer cells [20]. The suppression of MMP-13 by Rg3 plays a role in the inhibition of metastasis of melanoma cancer cells [21]. In addition, Rg3 inhibits the migration of colon cancer cells by suppressing the expression of nuclear factor-*κ*B- (NF-*κ*B-) regulated gene products, including MMP-9 [22]. What is more, Rg3 could downregulate the MMP9/MMP2 expression to inhibit vasculogenic mimicry in pancreatic cancer [30]. So, we hypothesized that Rg3 may inhibit the migration and invasion activity of NPC cells via downregulating MMPs expression. Our results showed that MMP-2 and MMP-9 mRNA and protein levels in NPC cells declined upon Rg3 stimulation. The influence of Rg3 on MMP-2 and MMP-9 expression identified in this study was in agreement with previous studies. Our results confirmed that Rg3 can suppress MMP-2 and MMP-9 expression in NPC cells.

EMT is a key step toward cancer invasion and metastasis. Several studies have demonstrated that EMT-related proteins in tumor tissues, especially the EMT markers, indicate a poor survival of NPC patients [31–33]. Then we evaluated whether Rg3 regulated EMT markers in NPC cells. The epithelial cells marker, E-cadherin, increased markedly upon Rg3 stimulation. On the contrary, Rg3 suppressed the expression of mesenchymal cells markers which include Vimentin and N-cadherin. As EMT is always induced by external stimulus, we next tested whether Rg3 is able to reverse TGF-*β*-induced EMT. NPC cells presented with typical morphological transition and unique proteins change of EMT under TGF-*β* stimulation. On the other hand, Rg3 attenuated these effects caused by TGF-*β*. It hinted that Rg3 can reverse EMT in NPC cells. Then we further investigated the mechanism of Rg3 regulating EMT. There is a theory that Rg3 can direct at multiple targets to treat tumors [8]. Rg3 inhibits cyclooxygenase-2 (COX-2) expression and NF-*κ*B activation to induce the apoptosis of breast cancer cells [34]. Rg3 enhances p53 acetylation to suppress the proliferation of melanoma cells [35]. Besides, Rg3 restrains angiogenesis by inhibiting phosphoinositide 3-kinase (PI3K) and extracellular signal regulated kinase 1/2 (ERK1/2) signal pathways [36]. What is more, Rg3 is an important inhibitory EMT agent by targeting FUT4 in lung cancer [37]. In this study, we detected the change of EMT-TFs upon Rg3 stimulation. We revealed that Rg3 is able to hamper the expression of EMT-TFs at different levels. These data suggested that Rg3 may suppress EMT in NPC cells through targeting EMT-TFs.

In summary, we have demonstrated that Rg3 is able to inhibit the migration and invasion of NPC cells. The effect of Rg3 on NPC migration and invasion may partly result from the downregulation of MMP-2 and MMP-9 expression. In addition, Rg3 can reverse the process of EMT and hamper the expression of EMT-TFs. Suppressing EMT may be another mechanism for Rg3 to inhibit NPC migration and invasion. However, there are certain limitations in the present study. Firstly, we did not conduct researches at the tumor tissues level. Besides, in vivo experiments were also needed to validate the effect of Rg3. Further deeper researches may update and verify our results.

## 5. Conclusion

Our study demonstrated that ginsenoside Rg3 could inhibit migration and invasion of NPC cells and suppress EMT. Rg3 is a promising agent for preventing and inhibiting NPC metastasis.

## Data Availability

The data used to support the findings of this study are available from the corresponding author upon request.
